# Eletriptan provides consistent migraine relief: results of a within-patient multiple-dose study

**DOI:** 10.1186/1129-2377-14-S1-P131

**Published:** 2013-02-21

**Authors:** M Almas, S Tepper, S Landy, E Ramos

**Affiliations:** 1 Pfizer Inc, USA; 2 Cleveland Clinic, USA; 3 Wesley Neurology and Headache Clinic, USA

## Objective

To evaluate the consistency of response to eletriptan in migraine.

## Background

Surveys indicate that more than 40% of individuals have an average headache frequency of 2 or more attacks per month. Lack of consistent response has been cited in patient surveys as one of the top 3 reasons for dissatisfaction with migraine treatments.

## Methods

Patients first completed an open-label, lead-in period in which they treated 3 migraine attacks with eletriptan 40 mg. Based on response to open-label treatment, patients were treated with either eletriptan 40 mg (E40; N=539) or eletriptan 80 mg (E80; N=432) in a 4-attack consistency of response study in which placebo was substituted, in a randomized, double-blind fashion, for the treatment of one attack. Headache response was defined as improvement at 2 hours post-dose to a headache intensity of none (pain-free) or mild. Within-patient consistency was defined a priori as headache response at 2 hours on at least 2 out of 3 attacks.

## Results

During double-blind treatment, within-patient consistency was 77% for E40 and 73% for E80. For patients (N=47) who had responded to 0/3 attacks on E40 in the open-label phase, titration to the E80 dose resulted in headache response in 55%. A repeated measures logistic regression analysis found that sustained headache response at 24 hours, averaged across 3 attacks, was significantly higher on both E40 vs. placebo (49% vs. 32%; p < 0.001) and E80 vs. placebo (43% vs. 11%; p < 0.001). Sustained pain-free at 24 hours, averaged across all 3 attacks, was also significantly higher on both E40 vs. placebo (30% vs. 5%; p < 0.001) and E80 vs. placebo (25% vs. 3%; p < 0.001).

## Conclusion

Eletriptan showed consistent and sustained efficacy in the treatment of migraine. Funded by Pfizer Inc.

